# 5G Traffic Prediction Based on Deep Learning

**DOI:** 10.1155/2022/3174530

**Published:** 2022-06-24

**Authors:** Zihang Gao

**Affiliations:** Department of Information and Technology, Wenzhou Vocational College of Science and Technology, Wenzhou 325006, China

## Abstract

The demand of wireless access users is increasing explosively. The 5G network traffic is increasing exponentially and showing a trend of diversity and heterogeneity, which makes network traffic forecasting face many challenges. By studying the actual performance of the 5G network, this paper makes an accurate prediction of the 5G network and builds a smoothed long short-term memory (SLSTM) traffic prediction model. The model updates the number of layers and hidden units according to the prediction accuracy adaptive mechanism. At the same time, in order to reduce the randomness of the 5G traffic sequence, the output feature sequence of the original time series is stabilized by the seasonal time difference method. In the experiments, the prediction results of the proposed algorithm are compared with those of the traditional algorithms. The results show that the SLSTM algorithm can effectively improve the accuracy of 5G traffic prediction. The model can be used for 5G traffic prediction for decision-making.

## 1. Introduction

With the rapid deployment and development of the Internet of Things (IoT), the 4G network is facing the challenge of nearly a thousand times the mobile traffic and has gradually been unable to meet the demand for massive access to the IoT. The 5G technology is developed and used to meet the explosion of mobile communication needs. The new technology revolution of rapid growth has vigorously promoted the digitization, networking, and intelligence of the economy and society [1–4]. The 5G network has the advantages of ultra-high speed, ultra-reliability, and ultra-short delay. It can be accessed anytime and anywhere, which meets the resource requirements of massive terminal access. However, it is accompanied by exponential growth of network traffic, diversity, and heterogeneity. In order to solve the huge traffic load caused by massive heterogeneous data on traditional cellular networks, 5G operators deploy a large number of low-power micro-base stations and pico-base stations on the periphery of macro-base stations to offload traffic and achieve load balancing for macro-base stations. At the same time, in order to optimize the deployment and allocation of 5G cellular network resources in large-scale cities and improve the intelligence and reliability of traffic management, it is crucial to predict traffic with high accuracy. 5G network traffic is essentially time-series data, so its prediction problem can be transformed into a time-series prediction modeling problem. Existing research mainly focuses on two categories of methods: parametric and nonparametric model methods [5–7]. The former methods mainly model and predict traffic flow based on mathematical theoretical knowledge such as statistics and probability distribution. This type of method models traffic flow through finite parameters and does not depend on the size of the dataset. Reference [8] analyzed the network traffic of many cellular base stations and distinguished the traffic into two parts, i.e., predictable and unpredictable ones, which proves that the predictable traffic has autocorrelation. Reference [9] proposed a seasonal SARIMA model, which accurately captured the seasonal characteristics of network traffic by analyzing the autocorrelation of time series, and then obtained long-term traffic forecast results. In order to verify whether the traffic of the cellular base station is affected by the number of base stations in the surrounding area, reference [10] proposed a model to predict network traffic from the perspective of time and space. The experimental results showed that the prediction accuracy of this model is significantly improved compared with the previous linear frameworks. As cellular networks expanded in width and depth, the network traffic characteristics have long deviates from the linear prediction models described above. Although nonlinear prediction models such as the generalized multifractal wavelet model and the autoregressive conditional heteroscedasticity model can describe the nonlinear characteristics of flow [11–14], their parameter estimation and model fitting have the problem of low accuracy. In recent years, with the help of the rapid development of big data collection technology and artificial intelligence technology, deep learning has gradually become a popular direction of nonparametric prediction models and has been favored by more researchers. Early shallow learning methods such as the support vector regression (SVR) model can better solve the learning problem of small sample traffic data, but cannot rely on comparative experiments or exhaustive search to obtain model parameters of large sample traffic data, which seriously affects learning ability and generalization ability [15–17]. On the other hand, the shallow learning methods are easy to capture the temporal correlation of network traffic, but not easy to capture the spatial characteristics. The multilayer architecture makes the 5G network traffic depend on heterogeneity in the spatiotemporal dimension, and it is necessary to capture the spatiotemporal dimension features at the same time to improve the traffic prediction performance. Therefore, how to accurately predict large-scale and high-complexity 5G network traffic with the powerful learning ability of deep learning has become one of the urgent problems for operators to efficiently supervise cellular networks and improve user service quality [18–22]. Based on the convolutional neural network (CNN), literature [18] designed the XGBoost model for traffic prediction, and the experiments proved that CNN can effectively extract the spatial features of traffic. Reference [19] combined CNN and the long short-term memory network (LSTM) to form a Conv-LSTM module, which effectively reduced the prediction error by extracting the spatiotemporal correlation of traffic. Reference [20] predicted network traffic based on a hybrid deep learning model of LSTM and stacked autoencoder (SAE). For 5G traffic flow prediction methods mentioned above, more complex models are used to improve the accuracy of prediction. And the prediction effect is rarely improved by processing eigenvalues. Therefore, for the adaptive ability of 5G traffic prediction, this paper uses the method of data autocorrelation analysis to preprocess the data sequence and proposes a 5G traffic prediction model based on smooth LSTM (SLSTM). The proposed method uses the relevant data to conduct experiments and analysis. By comparing and analyzing a large number of experimental results with some existing algorithms [9–19], it is found that the proposed model has the advantages of small calculation amount and good real-time performance in 5G traffic prediction. The higher tolerance can not only effectively improve the prediction accuracy but also be simpler and more efficient than other algorithms. The model updates the number of layers and hidden units according to the prediction accuracy adaptive mechanism. At the same time, in order to reduce the randomness of the 5G traffic sequence, the output feature sequence of the original time series is stabilized by the seasonal time difference method. In the experiments, the prediction results of the proposed algorithm are compared with those of the traditional algorithm. The results show that the SLSTM algorithm can effectively improve the accuracy of 5G traffic prediction.

## 2. Method Description

### 2.1. LSTM

**Figure 1:**
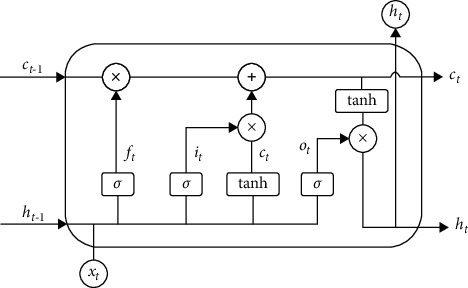
Basic structure of LSTM.

The LSTM network is an improvement of the recurrent neural networks (RNN) algorithm [19–21]. A cell is added to the original algorithm to increase the long-term memory function, so that the information is no longer attenuated, so as to overcome the problem of gradient disappearance in the RNN network. In the LSTM network, the added cells are usually composed of three threshold structures of forget gate, input gate, and output gate and a state vector transmission line. Among them, the state vector transmission line is responsible for long-term memory and the three thresholds are responsible for the selection of short-term memory. The forget gate determines how to retain the historical information of the memory module at the current moment. The input gate determines the transmission of input layer information to the hidden layer memory module. The output gate determines the memory and output of module information. When the information enters the LSTM network, it is judged whether it is useful or not according to the set rules, and the information that is judged to be useful is left. The rest of the information is forgotten through the forget gate. The schematic diagram of the LSTM network model is shown in Figure 1, in which the rectangular box represents the neural network layer and the circular box represents the point-by-point operation.

The forward propagation process of information in the network is described as follows:

Step 1: the forget gate is updated. The information that is allowed to pass through the cell is selected by the sigmoid neural layer. The input values *h*_*t*−1_ and *x*_*t*_ are input to the sigmoid function, and output a vector *f*_*t*_ with a value of 0∼1, indicating the proportion of each part of the information passing, where 1 means all information is passed and 0 means all information is discarded. The functional relationship between the output vector *f*_*t*_ and the input value *h*_*t*−1_ and *x*_*t*_ is as follows:
  (1) $$\begin{array}{c} f_{t}=\sigma \left(W_{f}\cdot \left[h_{t-1},x_{t}\right]+b_{f}\right), \end{array}$$
where *W*_*f*_ and *b*_*f*_ represent the weight and bias of the forget gate, respectively, and *σ* represents the sigmoid function.
Step 2: the cell status value is updated. This process decides what information needs to be updated and replaces the initial state value with an alternative value generated by the tanh layer.
The intermediate vectors *i*_*t*_ and ${\tilde{C}}_{t}$ are related with the input vector *h*_*t*−1_ and *x*_*t*_, which are described as follows:
  (2) $$\begin{array}{c} i_{t}=\sigma \left(W_{i}\cdot \left[h_{t-1},x_{t}\right]+b_{i}\right),\\ {\tilde{C}}_{t}=\tanh\left(W_{C}\cdot \left[h_{t-1},x_{t}\right]+b_{C}\right). \end{array}$$
Then, the state *C*_*t*−1_ to state *C*_*t*_ are updated, that is, multiplying *C*_*t*−1_ by the proportion of the information passed *f*_*t*_, and then discard the information according to proportion. Among them, the update equation of the state *C*_*t*_ is as follows:
  (3) $$\begin{array}{c} C_{t}=f_{t}\cdot C_{t-1}+i_{t}\cdot {\tilde{C}}_{t}. \end{array}$$
Step 3: the output value is determined. First, the part of information to output through the sigmoid layer is determined. Then, the state vector through the tanh layer is processed, and it is multiplied with the output weight of the sigmoid layer to get the final result. The intermediate vector *O*_*t*_, input vector *h*_*t*−1_ and *x*_*t*_, and output value *h*_*t*_ are related as follows:
  (4) $$\begin{array}{c} O_{t}=\sigma \left(W_{o}\cdot \left[h_{t-1},x_{t}\right]+b_{O}\right).\\ h_{t}=O_{t}\times \;\tanh\;C_{t}, \end{array}$$
where *W*_*o*_ and *b*_*O*_ are the weight and bias of the output gate, respectively.

The LSTM network also includes a backpropagation process, starting from the current time *t*, calculating the error term at each time, and propagating it to the upper layer. The gradient of each weight is calculated according to the corresponding error term, and the parameters are iteratively updated by gradient descent.

### 2.2. SLSTM for 5G Traffic Prediction

Since 5G traffic has the characteristics of dynamic change with time, LSTM is suitable for the analysis and prediction of 5G traffic data. The 5G traffic data time series is a random time series, which usually shows nonstationary characteristics. Therefore, it is necessary to evaluate the stationarity of the original 5G traffic observation series. Here, auto correlation function (ACF) is used to obtain the estimated value.

(5) $$\begin{array}{c} \mathrm{ACF}=\frac{\sum_{t=k+1}^{n}\left[\left(X_{t}-\bar{X}\right)\left(X_{t-k}-\bar{X}\right)\right]}{\sum_{t=1}^{n}{\left(X_{t}-\bar{X}\right)}^{2}}. \end{array}$$

The ACF chart of the estimated value is drawn to judge the state of the time series and confirm the period. When the autocorrelation function value exceeds the 95% confidence interval and there is an obvious tailing effect, the series can be judged as a nonstationary series. Otherwise, the series is a stable sequence.

When the 5G traffic sequence is a nonstationary sequence, its periodic difference ∇^*T*^*X*_*t*_ can be calculated based on the original traffic sequence to obtain a stationary difference sequence {∇^*T*^*X*_*t*_, *t*=1,2,3,…}. Here, the stationary difference sequence is used as the output field of the sample set to reconstruct the sample set. The SLSTM is used to predict the result, and restore the result to the original 5G traffic.

**Figure 2:**
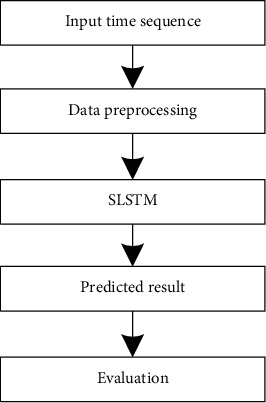
Prediction of 5G traffic based on SLSTM.

Figure 2 shows the general flow of the SLSTM algorithm implementation. It can be seen that the algorithm is divided into two processes: forward propagation and backward propagation. The forward propagation is mainly the calculation of the training results of the input training samples, and the backward propagation is the reverse update of network weights and biases. The SLSTM algorithm implemented in this paper adds a data stabilization process before the forward propagation process on the basis of the above process and adds a data sequence restoration process after outputting the prediction results.

## 3. Experiment and Analysis

### 3.1. Dataset and Preprocessing

The dataset used in this experiment is from a foreign network operator. The dataset is generated in two modes: static and in-vehicle in the 5G network environment. Large bandwidth is considered to be one of the remarkable characteristics of 5G networks. This paper uses traffic bandwidth data as an indicator to measure the performance of 5G networks. The traffic downlink bandwidth data generated by file download in a static environment is used as the input data, and the CNN, gate recurrent unit (GRU), and LSTM models are selected for comparison with the model in this paper. First, the training data is preprocessed. The original dataset is multiple small files, and the data collected every 5 days during the experimental period is integrated. Then, some useless data is removed, and some data that is not in the downloading state is given up, that is, the data whose download bandwidth is 0 (this part of invalid data is considered to be interference caused by the device or the environment). Finally, the 5G network bandwidth traffic characteristics of the dataset are analyzed. The input characteristics of bandwidth prediction are determined. The bandwidth data and time data are normalized to between (0, 1), which are used as the input of the method in this paper.

### 3.2. Evaluation Index

In order to better evaluate and analyze the prediction results of the network model, the root mean square error (RMSE), mean absolute error (MAE), and *R*-squared are used as evaluation indicators. The mathematical expressions of the evaluation formula are shown in formulas (6)–(8). The RMSE can more accurately reflect the degree of dispersion of the training model. When the value of RMSE is smaller, it indicates that the degree of aggregation of the model is higher, and the model is more accurate.

(6) $$\begin{array}{c} \text{RMSE}=\sqrt{\frac{1}{n}\sum_{i=1}^{n}{\left(f_{i}-y_{i}\right)}^{2}}. \end{array}$$

The MAE is to take the difference between the predicted result of the experiment and the actual result. And the absolute value is first used, and then the mean value is calculated. When the value of MAE is smaller, the error of the model is smaller.

(7) $$\begin{array}{c} \text{MAE}=\frac{1}{n}\sum_{i=1}^{n}\left|f_{i}-y_{i}\right|. \end{array}$$

R-squared is considered by scholars to be one of the best methods to measure linear regression. *R*-squared mainly converts the prediction results into the accuracy before the range of 0 to 1, which can more intuitively show the accuracy of a model. When the fit of the model is very ideal, its value will be wirelessly close to 1.

(8) $$\begin{array}{c} R^{2}=1-\frac{\sum_{i=1}^{n}{\left(y_{i}-f_{i}\right)}^{2}}{\sum_{i=1}^{n}{\left(y_{i}-\bar{y}\right)}^{2}}. \end{array}$$

### 3.3. Result and Discussion

For the training dataset, the LSTM, GRU, CNN, and SLSTM model proposed in this paper are used for experiments. The experimental results are shown in Table 1. The experimental results show that the training results of LSTM and GRU are close, and the difference of *R*-squared is only one percentage point. Compared with the method in this paper, the three types of comparison methods have gaps in performance under the three evaluation indicators. In particular, comparing the LSTM method with the improved model in this paper, the performance improvement of the method in this paper is more obvious, and the *R*-squared index is improved by 4%. These results prove the effectiveness of the proposed method.

**Figure 3:**
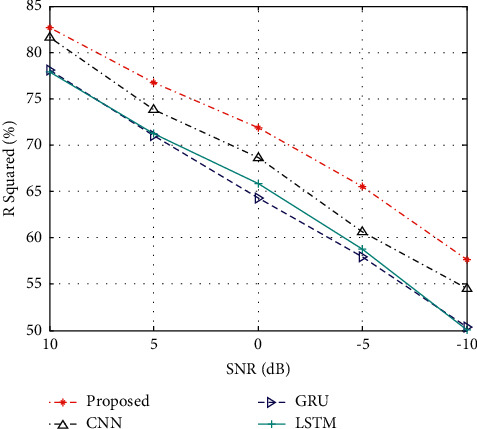
Prediction performance under noise corruption.

In the actual process, due to the influence of signal transmission factors, certain errors may occur in the prediction model. To this end, the experimental data is subjected to a certain degree of noise condition to reflect the volatility of flow data. On this basis, *R*-squared is used as the basic evaluation index to test the prediction performance trend of various methods, and the results are shown in Figure 3. It can be seen from the figure that the performance of various methods is degraded to a certain extent due to the influence of noise. In contrast, the method in this paper can maintain a more robust performance under different noise interference conditions, showing its advantages.

## 4. Conclusion

In the complex environment of 5G networks, high-precision traffic prediction is of great significance to the planning and scheduling of network resources and is conducive to reliable and efficient transmission of network data. In this paper, a 5G traffic prediction model based on the SLSTM is established, and the data sequence is preprocessed by the method of data autocorrelation analysis to further improve the reliability of the model. Analysis and verification of real data show that the model can effectively predict 5G traffic. Comparing the method proposed in this paper with several existing 5G traffic prediction methods, the results show that the model in this paper has higher fitting degree, smaller error, and more accurate prediction and has certain advantages. However, there is still room for improvement in this model. For example, the factors affecting 5G traffic are not considered comprehensively. In the follow-up research, it is necessary to consider related 5G traffic factors in multiple dimensions, so as to better adapt to the actual situation of 5G use.

## Figures and Tables

**Table 1:** Comparison of prediction performance of different methods.

	RMSE	MAE	R-squared
Proposed	3014.2	287.3	0.83
CNN	3054.3	289.6	0.81
GRU	3096.1	295.1	0.79
LSTM	3102.5	296.8	0.78

## Data Availability

The dataset can be accessed upon request.
